# Unified real-time environmental-epidemiological data for multiscale modeling of the COVID-19 pandemic

**DOI:** 10.1038/s41597-023-02276-y

**Published:** 2023-06-07

**Authors:** Hamada S. Badr, Benjamin F. Zaitchik, Gaige H. Kerr, Nhat-Lan H. Nguyen, Yen-Ting Chen, Patrick Hinson, Josh M. Colston, Margaret N. Kosek, Ensheng Dong, Hongru Du, Maximilian Marshall, Kristen Nixon, Arash Mohegh, Daniel L. Goldberg, Susan C. Anenberg, Lauren M. Gardner

**Affiliations:** 1 Department of Civil and Systems Engineering, Johns Hopkins University, Baltimore, MD 21218 USA; 2 Department of Earth and Planetary Sciences, Johns Hopkins University, Baltimore, MD 21218 USA; 3 Department of Environmental and Occupational Health, Milken Institute School of Public Health, George Washington University, Washington, DC 20052 USA; 4 College of Arts and Sciences, University of Virginia, Charlottesville, VA, 22903, USA; 5 Division of Infectious Diseases and International Health, University of Virginia School of Medicine, Charlottesville, VA 22903 USA; 6 Department of Emergency Medicine, Chi-Mei Medical Center, Tainan, Taiwan; 7 Present Address: Health & Exposure Assessment Branch, California Air Resources Board, Sacramento, CA 95812 USA

**Keywords:** Viral infection, Environmental impact, Climate sciences

## Abstract

An impressive number of COVID-19 data catalogs exist. However, none are fully optimized for data science applications. Inconsistent naming and data conventions, uneven quality control, and lack of alignment between disease data and potential predictors pose barriers to robust modeling and analysis. To address this gap, we generated a unified dataset that integrates and implements quality checks of the data from numerous leading sources of COVID-19 epidemiological and environmental data. We use a globally consistent hierarchy of administrative units to facilitate analysis within and across countries. The dataset applies this unified hierarchy to align COVID-19 epidemiological data with a number of other data types relevant to understanding and predicting COVID-19 risk, including hydrometeorological data, air quality, information on COVID-19 control policies, vaccine data, and key demographic characteristics.

## Background & Summary

The ongoing COVID-19 pandemic has caused widespread illness, loss of life, and societal upheaval across the globe. As the public health crisis continues, there is both an urgent need and a unique opportunity to track and characterize the spread of the virus. This includes improving our understanding of the spatiotemporal sensitivity of disease transmission to demographic, geographic, socio-political, seasonal and environmental factors.

The global research and data science communities have responded to this challenge with a wide array of efforts to collect, catalog, and disseminate data on COVID-19 case counts, hospitalizations, mortality, vaccinations, and other indicators of COVID incidence and burden^1–14^. While these databases have supported a tremendous volume of research, risk monitoring, and public discussion, they often have inconsistent structure, naming conventions, values, resolution, quality, and lack alignment between infectious disease data and the potential risk factors. These issues require laborious cleanup to combine data from different sources that delays research progress and may affect its quality. Additionally, critical datasets that quantify risk factors such as climate and human mobility are subject to biases and limited availability, posing further challenges for data processing.

To utilize these disparate types of data from different sources at different levels of granularity, they need to be combined and harmonized. Without proper harmonization, curation, and consistency checks, analyzing these datasets can lead to spurious results. A unified dataset that addresses these issues will help to accelerate our understanding of COVID-19 risk through multiscale spatiotemporal modeling by eliminating the extra time-consuming steps needed to clean, standardize, and merge the different data sources. As an example, we provide a test case with generating estimates of effective reproductive number (R_t_) from two different data sources, including reported case counts and estimated daily infections, that are directly imported from our unified dataset without consuming time on unifying the variable names/types and cleaning or georeferencing the data.

Thus, our *Unified COVID-19 Dataset* aims to (1) harmonize naming and coding conventions from credible data sources at multiple administrative levels, (2) implement quality control for COVID-19 case counts of different types, (3) systematically align potential predictors with COVID-19 data, and (4) provides real-time updates and corrections, and incorporates new sources for relevant variables as they become available. Specifically, the *Unified COVID-19 Dataset* set includes key components for epidemiology, including demography, hydrometeorology, air quality, policy, vaccination, and healthcare accessibility, maps all geospatial units globally into a unique identifier, standardizes administrative names, codes, dates, data types, and formats, unifies variable names, types, and categories. We also curate the data to correct for confusing entries that arise from the conflicting names of the same geographic units, different reporting strategies and schedules, and accumulation of epidemiological variables. The dataset is distributed in accessible formats, and optimized for machine learning applications to support reproducible research of high quality. The availability of this dataset has facilitated analyses of COVID-19 risk factors at subnational resolution across multiple countries^15–18^ and studies of changes in risk factors over the course of the pandemic^19^.

## Methods

We compile epidemiological data from different sources, translate the data records, and check the available case types. Then, the variable and unit names are standardized and geo-coded using a unified geospatial identifier (ID) to support aggregation at different administrative levels and consistent merging into a single time-varying epidemiological dataset file. The case types that are not included in the raw data are derived from the existing case types whenever possible (e.g., deriving active cases from confirmed cases, recoveries, and deaths). A lookup table provides key geographic names and codes while the static data fields, including air quality estimates, are combined in a separate dataset file. Time-varying hydrometeorological and policy data are processed to extract the variables and indices for each geospatial ID at a daily resolution. In accordance with FAIR data principles^20^, we adopt an approach through which the data are *findable* through a persistent DOI, appropriate metadata, and indexing, *accessible* as a free and open resource that can be retrieved through standard protocols, *interoperable* in the use of widely used data formats and structures, and *reusable* through the provision of licensing and provenance information and conformance with data standards.

### Data harmonization

**Fig. 1:**
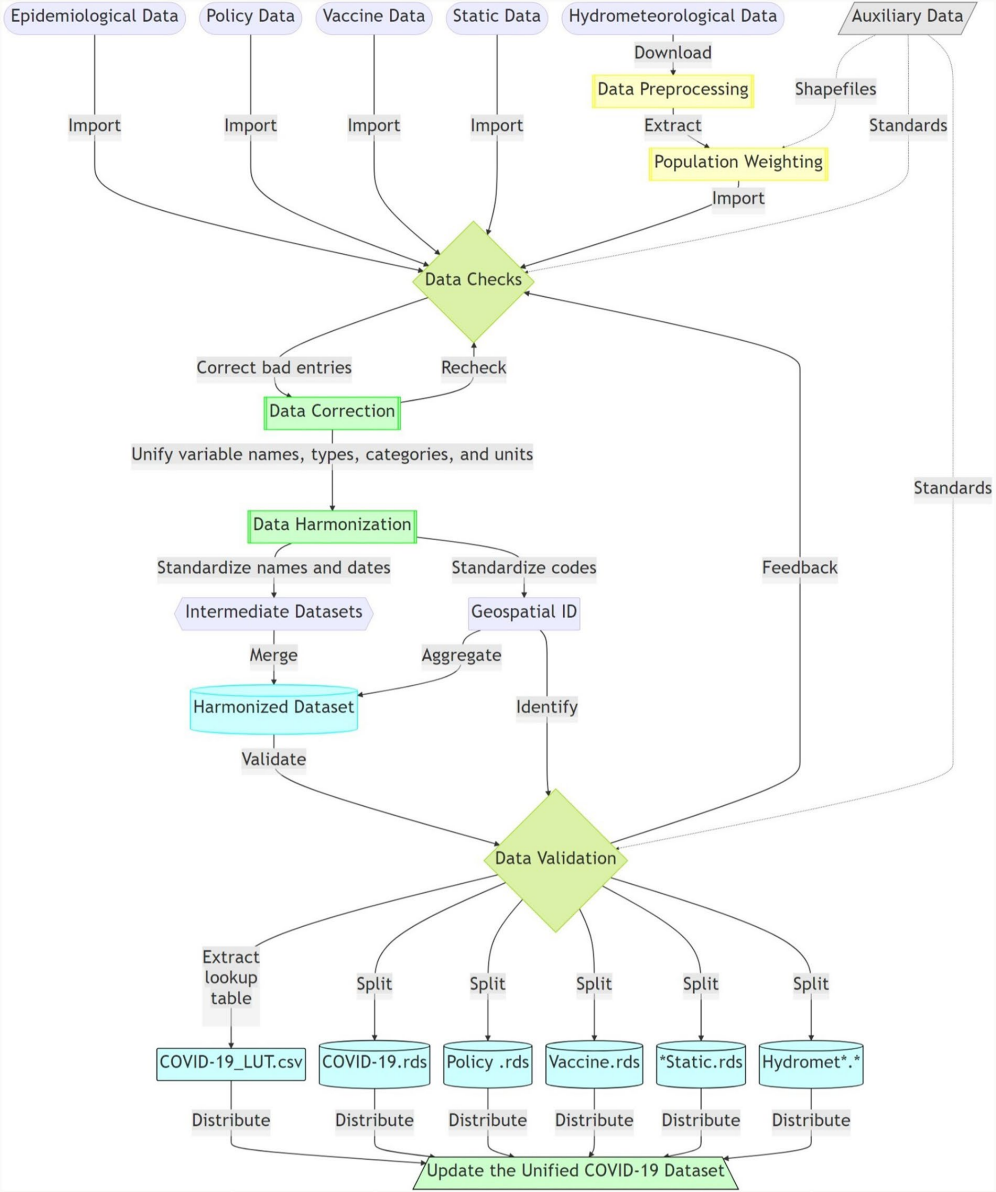
Flowchart of the data harmonization for the unified COVID-19 dataset.

The dataset follows the data harmonization flowchart, shown in Fig. 1, to integrate disparate multi-dimensional data across multiple types and resources. Multiple data types will require standardization, ranging from geospatial identification, variable type, variable name, and data structures. We map all geospatial units into a unique identifier. Each unit in the spatial datasets are mapped to a unique geospatial ID which in turn enables merging the datasets by the unified ID, together with other grouping factors such as data source, type, variable, time/date, and other dimensions. The national-level IDs are based on ISO 3166-1 alpha-2 codes, and subnational data use Federal Information Processing Standard (FIPS) codes (U.S.), Nomenclature of Territorial Units for Statistics (NUTS) codes (Europe), ISO 3166-2 codes (global provinces or states), and local identifiers (global administrative levels 2 and 3). This also standardizes administrative names, codes, dates, data types, and formats with unified variable names, ids, types, and categories as well as curates the data, link records, and eliminates ambiguity that arise from the conflicting names of the same geographic units and the different reporting strategies and schedules.

To georeference the data, we first use the IDs (identifiers or codes) and shapefiles, if available, from the original data sources to map standardized names in English language with UTF-8 encoding. We implement unification functions using standard conversions from the different coding systems (e.g., Nomenclature of Territorial Units for Statistics (NUTS) system for Europe, Official municipality key/Amtlicher Gemeindeschlüssel (AGS) for Germany, and Federal Information Processing Standard (FIPS) codes for the U.S. counties and states) and unit names into the unified geospatial ID system and address any ambiguous names of known duplicates of the same geographic unit, via built-in re-coding functions or lookup tables. Data validation and consistency checks are applied to ensure that the standardized names are mapped correctly and are consistent with the original names and geographic coordinates. If a geographic unit is split into smaller sub-regions, new IDs are assigned to the higher-resolution units. When the IDs and shapefiles are not provided in the initial dataset, the data will be merged by name, and manually mapped into unique identifiers. The unit names will be converted into standardized codes where problematic entries will be detected and manually inspected. The lookup table provides the standardized geographic names and codes, and the unification functions will be updated to address the known issues and re-coding exceptions. Additional approaches are implemented to harmonize the other dataset features such as variable type, variable name, and data structure.

### Geospatial ID

**Fig. 2:**
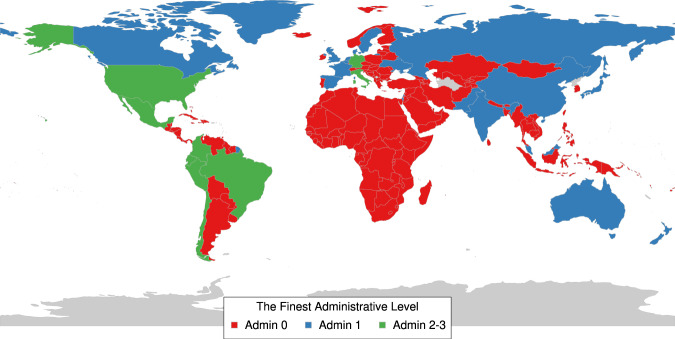
Spatial coverage map for the unified COVID-19 dataset (Admin 0 = National, Admin 1 = First administrative level (e.g., state, province), Admin 2–3 = Second and third administrative levels (e.g., county, district).

**Fig. 3:**
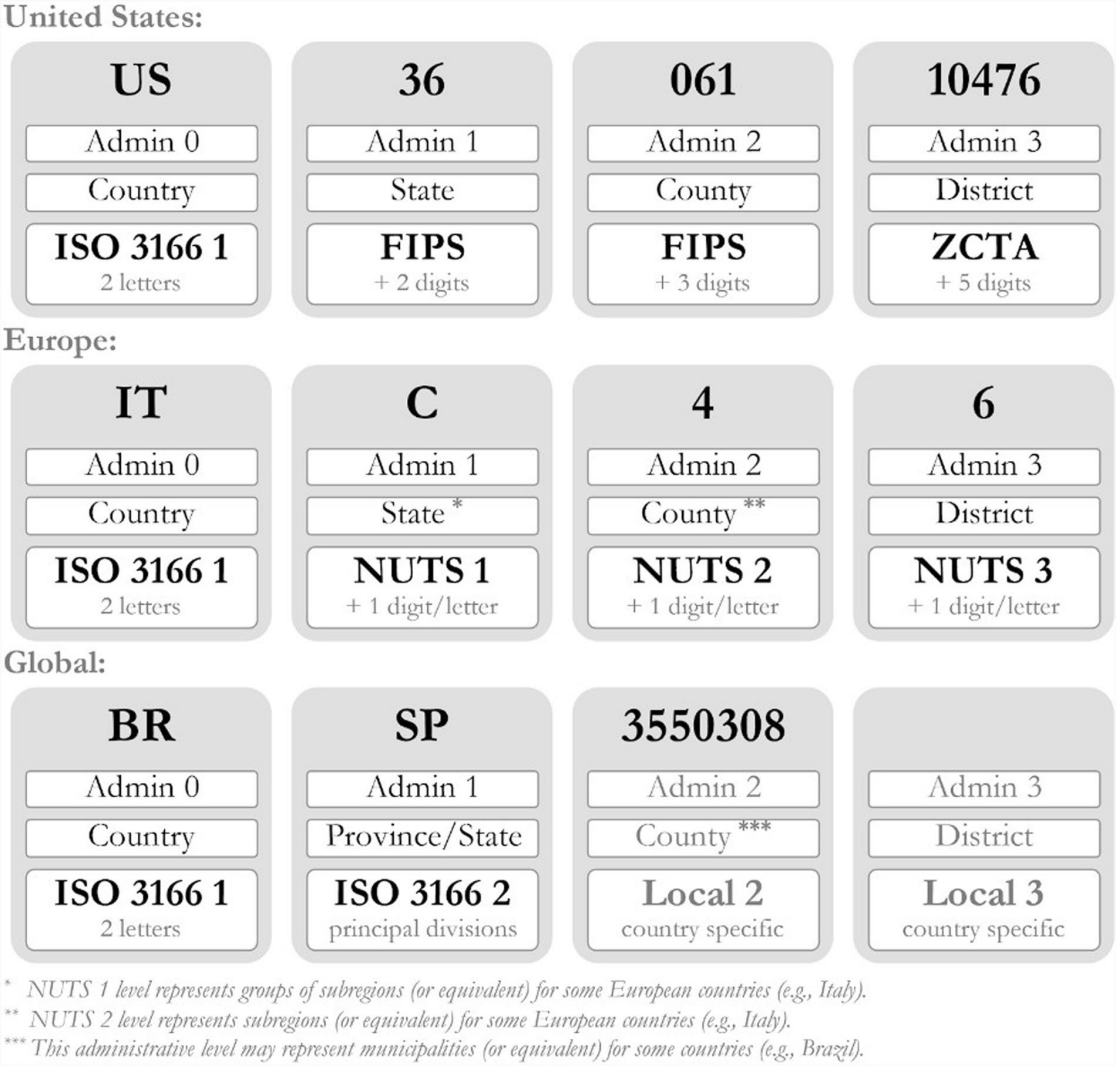
Geospatial ID used for the unified COVID-19 dataset.

The spatial coverage of the dataset is shown in the world map in Fig. 2 and the geospatial ID system is shown in Fig. 3. The national-level IDs are based on ISO 3166-1 alpha-2 codes. The subnational administrative levels for the United States (at the state and county levels) are based on the Federal Information Processing Standard (FIPS) codes. For Europe, all administrative levels use the Nomenclature of Territorial Units for Statistics (NUTS) codes. Globally, the principal subdivisions (e.g., provinces or states) use ISO 3166-2 codes while higher resolution units are based on local identifiers (e.g., for Brazil, municipalities use IBGE codes from the Brazilian Institute of Geography and Statistics).

### Update frequency

Most components are updated on a daily basis while time-consuming data extraction for hydrometeorological variables, with and without population weighting, are updated monthly. The dataset is disseminated through the Center for Systems Science and Engineering (CSSE) at Johns Hopkins University (JHU), the source of the widely accessed JHU Coronavirus Dashboard^1^.

## Data Records

Table 1 summarizes the lookup table keys with the different unit IDs, names, codes, centroid coordinates, and population. The full unified dataset is available on Zenodo^21^.

**Table 1:** Lookup table for the unified COVID-19 dataset.

Column	Type	Description
ID	Character	Geospatial ID, unique identifier (described above)
Level	Character	Geospatial level (e.g., Country, Province, District, NUTS 0-3)
ISO1_3N	Character	ISO 3166-1 numeric code, 3-digit, admin. level 0 (countries)
ISO1_3C	Character	ISO 3166-1 alpha-3 code, 3-letter, admin. level 0 (countries)
ISO1_2C	Character	ISO 3166-1 alpha-2 code, 2-letter, admin. level 0 (countries)
ISO2	Character	ISO 3166-2 code, principal subdivisions (e.g., provinces)
ISO2_UID	Character	ISO 3166-2 code, principal subdivisions, full unique ID
FIPS	Character	Federal Information Processing Standard (FIPS, United States)
NUTS	Character	Nomenclature of Territorial Units for Statistics (NUTS, Europe)
AGS	Character	Municipality key/Amtlicher Gemeindeschlüssel (Germany)
IBGE	Character	Brazilian municipality code
ZTCA	Character	ZIP Code Tabulation Area (ZCTA, United States)
Longitude	Double	Geospatial coordinate (centroid), east–west
Latitude	Double	Geospatial coordinate (centroid), north–south
Population	Integer	Total population of each geospatial unit
Admin	Integer	Administrative level (0–3)
Admin0	Character	Standard name of administrative level 0 (countries)
Admin1	Character	Standard name of admin. level 1 (e.g., provinces)
Admin2	Character	Standard name of admin. level 2 (e.g., counties)
Admin3	Character	Standard name of admin. level 3 (e.g., districts and ZTCA)
NameID	Character	Full name ID of combined admin. levels, unique identifier

### Epidemiological data

Daily COVID-19 case counts are taken from the different data sources, including CSSE’s JHU Coronavirus Dashboard, and georeferenced to the administrative units in which they were diagnosed^1–12^. We merge multiple data sources with different case types. This includes translating variable names from different languages, transforming different data formats (e.g., accumulating daily counts from RKI data for Germany), and checking the aggregated counts against all data sources. Table 2 lists the epidemiological data structure. Table 3 describes the different case types, including confirmed cases, deaths, hospitalizations, and testing results.

**Table 2:** COVID-19 data structure.

Column	Type	Description
ID	Character	Geospatial ID, unique identifier
Date	Date	Date of data record
Cases	Integer	Number of cumulative cases
Cases_New	Integer	Number of new daily cases
Type	Character	Type of the reported cases
Age	Character	Age group of the reported cases
Sex	Character	Sex/gender of the reported cases
Source	Character	Data source: JHU^1^, CTP^2^, NYC^3^, NYT^4^, SES^5^, DPC^6^, RKI^7^, JRC^8^, IHME^13^

**Table 3:** COVID-19 case types.

Type	Description
Active	Active cases
Confirmed	Confirmed cases
Deaths	Deaths
Home_Confinement	Home confinement/isolation
Hospitalized	Total hospitalized cases excluding intensive care units
Hospitalized_Now	Currently hospitalized cases excluding intensive care units
Hospitalized_Sym	Symptomatic hospitalized cases excluding intensive care units
ICU	Total cases in intensive care units
ICU_Now	Currently in intensive care units
Infections	Estimated Infections
Negative	Negative tests
Pending	Pending tests
Positive	Positive tests, including hospitalised cases and home confinement
Positive_Dx	Positive cases emerged from clinical activity/diagnostics
Positive_Sc	Positive cases emerging from surveys and tests
Recovered	Recovered cases
Tested	Cases tested = Tests - Pending
Tests	Total performed tests
Ventilator	Total cases receiving mechanical ventilation
Ventilator_Now	Currently receiving mechanical ventilation

#### Epidemiological estimates

**Fig. 4:**
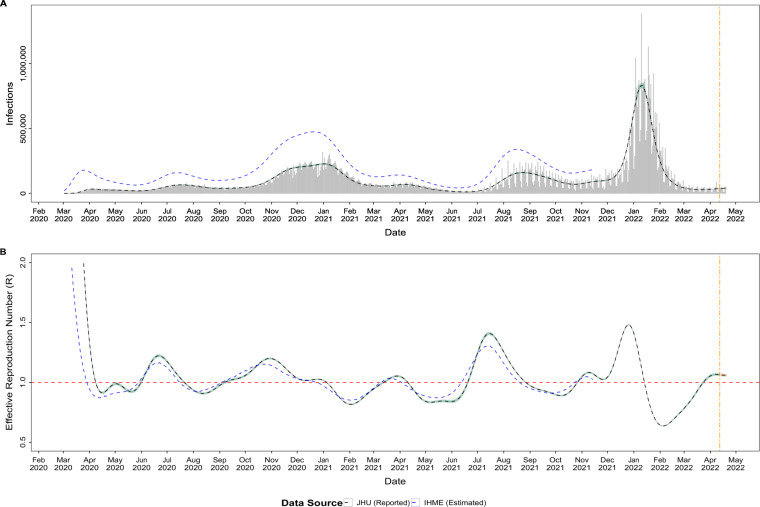
Epidemiological estimates and the reported COVID-19 cases for the USA. (**A**) Estimated daily infections (dashed lines) and the reported cases (vertical bars); (**B**) Effective reproduction number (R) estimated from the estimated of daily infections.

To facilitate analysis of reporting issues, such as underreporting and testing capacity limitations, we also integrated estimated daily infections from the Institute for Health Metrics and Evaluation (IHME)^13^. Fig. 4 shows a comparison of epidemiological estimates of daily infections and the reported COVID-19 cases as well as the corresponding effective reproduction number (R_t_) estimates for the USA. This is also an example of utilizing the harmonized COVID-19 data in our unified dataset for analysis and epidemiological estimates across different data sources that could use inconsistent location names and identifiers. The epidemiological estimates (cases by infection date and R_t_) are provided with the dataset for the United States at both national and state levels. Those estimates are generated using *EpiNow2* and *EpiEstim* R packages^14,22,23^. *EpiEstim* accounts for uncertainty in the mean and standard deviation of the generation interval by resampling over a range of plausible values. *EpiNow2* uses a Bayesian approach that also accounts for reporting delays. The parameters required for R_t_ estimates, specifically the distributions of incubation period and serial interval, are obtained from the literature^24–28^.

### Vaccination data

Global and US vaccine data are harmonized and integrated from the Johns Hopkins Centers for Civic Impact for the Coronavirus Resource Center (CRC)^29^. Cumulative numbers of people fully or partially vaccinated are provided by vaccine provided, whenever available, and dose types – including doses administered (in general or as first or second dose), allocated, or shipped/arrived to vax sites. Table 4 describes the data structure of the harmonized version of the vaccine dataset while Table 5 lists the different dose types.

**Table 4:** Vaccine Data Structure.

Column	Type	Description
**ID**	Character	Geospatial ID, unique identifier
**Date**	Date	Date of data record
**Vaccine**	Character	Common name of the vaccine provider, or all
**DoseType**	Character	Type of the vaccine dose
**DoseValue**	Double	Cumulative number of doses
**Vax_Full**	Double	Cumulative number of people fully vaccinated
**Vax_Partial**	Double	Cumulative number of people partially vaccinated

**Table 5:** Dose Types.

Type	Description
**Admin**	Doses administered
**Alloc**	Doses allocated
**Ship**	Doses shipped/arrived to vax sites
**Stage1**	Doses administered as first
**Stage2**	Doses administered as second

### Hydrometeorological data

Like many viral diseases, the stability of aerosolized SARS-CoV-2 and COVID-19 transmission are sensitive to hydrometeorological conditions. Human behavior and social interactions, dominant drivers of COVID-19 transmission, are also inextricably connected to local hydrometeorological conditions. For these reasons, the ability of this unified dataset to characterize spatiotemporal variations in hydrometeorological variables is germane to understanding COVID-19 transmission. Numerous studies have found relationships between meteorology and COVID-19 transmission rates^30–33^. As these studies demonstrate, however, the identified relationships are not always consistent across studies^34^, there may be differences in meteorological influence across different regions or stages of the pandemic, and the relative importance of hydrometeorological influence in impacting broad epidemiological trends is uncertain. Large, gridded hydrometeorological datasets can be challenging for non-experts to work with, and simpler weather station data are not always representative across large geographic units.

**Fig. 5:**
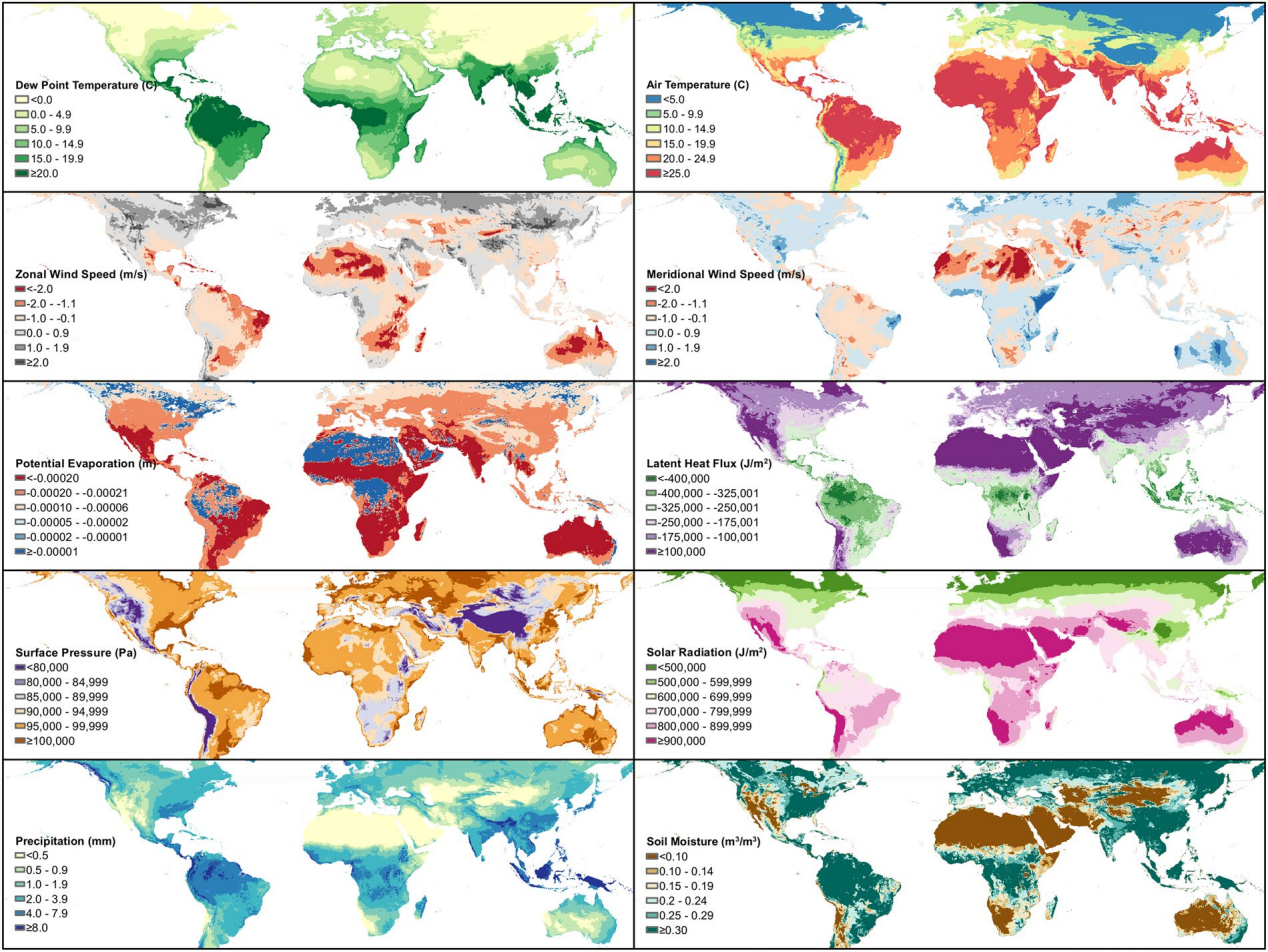
Global geographical distribution of the 10 hydrometeorological variables included in the dataset – average of all daily values for 2020.

To facilitate studies that integrate hydrometeorology to COVID-19 prediction, we include multiple hydrometeorological variables in our unified dataset. Table 6 lists the hydrometeorological variables extracted from NLDAS-2 and ERA5 while Fig. 5 shows maps of the 2020 averages. Population weighting is applied to gridded environmental data (hydrometeorology and air quality) to account for variation in the spatial distribution of the exposed human population within each unit. Gridded Population of the World v4 (GPWv4) population count data with adjustment to match United Nations estimates are obtained from the Center for International Earth Science Information Network (CIESIN) Socioeconomic Data and Applications Center SEDAC^35^. These counts are then applied as weights by calculating the fraction of the population within each unit at each level of the administrative hierarchy contained in each grid cell, multiplying gridded environmental variables by this fraction, and summing for the administrative unit. We derive these variables from the second generation North American Land Data Assimilation System (NLDAS-2), using the NLDAS-2 meteorological forcings and Noah Land Surface Model simulated surface hydrological fields, and the fifth generation European Centre for Medium-Range Weather Forecasts (ECMWF) atmospheric reanalysis of the global climate (ERA5)^36,37^. Both ERA5 and NLDAS assimilate observations and model output to provide continuous maps of meteorological variables without gaps or missing values in the data, which cannot be achieved from observations alone. The fine spatial resolution of NLDAS (0.125° latitude × 0.125° longitude) and ERA5 (0.25° latitude × 0.25° longitude) represents significant improvements over earlier datasets, and both datasets have been extensively tested against observations and found to capture the observed quantities^36–38^. ERA5 and NLDAS are available with a 4–6-day latency making these datasets particularly well-suited for forecasting COVID-19 dynamics in near real-time. NLDAS is available only for the contiguous United States, while ERA5 is available globally.

**Table 6:** Hydrometeorological data structure.

Column	Unit	Description
ID		Geospatial ID, unique identifier (described above)
Date		Date of data record
T	°C	Daily average near-surface air temperature (NLDAS^36^, ERA5^37^)
Tmax	°C	Daily maximum near-surface air temperature (NLDAS^36^, ERA5^37^)
Tmin	°C	Daily minimum near-surface air temperature (NLDAS^36^, ERA5^37^)
Td	°C	Daily average dew point temperature (NLDAS^36^, ERA5^37^)
Tdd	°C	Daily average dew point depression (NLDAS^36^, ERA5^37^)
RH	%	Daily average relative humidity (NLDAS^36^, ERA5^37^)
SH	kg/kg	Daily average specific humidity (NLDAS^36^, ERA5^37^)
MA	%	Daily average moisture availability (NLDAS^36^)
RZSM	kg/m2	Daily average root zone soil moisture content (NLDAS^36^)
SM	kg/m2	Daily average soil moisture content (NLDAS^36^)
SM1	m3/m3	Daily average volumetric soil water layer 1 (ERA5^37^)
SM2	m3/m3	Daily average volumetric soil water layer 2 (ERA5^37^)
SM3	m3/m3	Daily average volumetric soil water layer 3 (ERA5^37^)
SM4	m3/m3	Daily average volumetric soil water layer 4 (ERA5^37^)
SP	Pa	Daily average surface pressure (NLDAS^36^, ERA5^37^)
SR	J/m2	Daily average surface downward solar radiation (ERA5^37^)
SRL	W/m2	Daily average downward longwave radiation flux (NLDAS^36^)
SRS	W/m2	Daily average downward shortwave radiation flux (NLDAS^36^)
LH	J/m2	Daily average surface latent heat flux (ERA5^37^)
LHF	W/m2	Daily average surface latent heat flux (NLDAS^36^)
PE	m	Daily average evapotranspiration (ERA5^37^)
PEF	W/m2	Daily average potential evaporation (NLDAS^36^)
P	mm/day	Daily total precipitation (NLDAS^36^ and ERA5^37^)
U	m/s	Daily average 10-m Zonal wind speed (NLDAS^36^, ERA5^37^)
V	m/s	Daily average 10-m Meridional wind speed (NLDAS^36^, ERA5^37^)
Source		Data source: ERA5, NLDAS ± CIESIN^35^–^37^

We obtain gridded hourly ERA5 and NLDAS data for January 1, 2020 onwards. Hourly data are transformed to daily mean, maximum, minimum, or total values, depending on the variable. A land-sea mask is applied to the hydrometeorological data such that any water grid cells are excluded from the analysis. Two types of average values are provided for each administrative unit: simple averages and population-weighted averages. A small number of administrative units do not contain ERA5 or NLDAS grid cells due to their having irregular boundaries or small areal extents (e.g., ~15% of NUTS 3 divisions). In this case, we estimate the value of meteorological values at the unit’s geographic centroid using an inverse distance weighting interpolation method and thereafter calculate the simple and population-weighted averages using these interpolated values.

### Air quality data

Long-term exposure to air pollutants may increase susceptibility to severe COVID-19 outcomes^39–41^. We provide long-term averages of surface-level annual average nitrogen dioxide (NO_2_) and fine particulate matter (PM_2.5_) to allow this potential impact to be incorporated into studies. We use a dataset that observations of aerosol optical depth (AOD) from Earth-observing satellites to global estimates of surface-level PM_2.5_ using geophysical relationships between modeled PM_2.5_ and AOD from a chemical transport model and a Geographically Weighted Regression technique^42^. Global NO_2_ estimates are derived by scaling the predicted concentrations from a global land use regression model with annual satellite observations of tropospheric NO_2_ columns from the Ozone Monitoring Instrument satellite^43–45^.

PM_2.5_ and NO_2_ datasets are regridded from their native resolutions (0.01° latitude × 0.01° longitude and 1 km × 1 km, respectively) to 0.05° latitude × 0.05° longitude and averaged over 2014–2018. We calculate both simple and population-weighted averages of PM_2.5_ and NO_2_ for administrative units.

### Policy data

The time-varying policy response data described in Table 7 are processed from the Oxford COVID-19 Government Response Tracker (OxCGRT) for the policy types listed in Table 8, including four categories of policies: (i) **containment and closure policies:** C1: School closing, C2: Workplace closing, C3: Cancel public events, C4: Restrictions on gatherings, C5: Close public transport, C6: Stay at home requirements, C7: Restrictions on internal movement, and C8: International travel controls, (ii) **economic policies:** E1: Income support, E2: Debt/contract relief, E3: Fiscal measures, and E4: International support, (iii) **health system policies:** H1: Public information campaigns, H2: Testing policy, H3: Contact tracing, H4: Emergency investment in healthcare, H5: Investment in vaccines, H6: Facial Coverings, H7: Vaccination Policy, and H8: Protection of elderly people, and (iv) **miscellaneous policies:** M1: Wildcard as well as policy indices for containment health, economic support, and government response. The policies are differentiated depending on whether they apply to everyone (E policy type suffix), non-vaccinated people (NV policy type suffix), vaccinated people (V policy type suffix), or to the majority (M policy type suffix). For more details, see OxCGRT’s codebook, index methodology, interpretation guide, and subnational interpretation^46^.

**Table 7:** Policy data structure.

Column	Type	Description
ID	Character	Geospatial ID, unique identifier
Date	Date	Date of data record
PolicyType	Character	Type of the policy
PolicyValue	Double	Value of the policy
PolicyFlag	Logical	Logical flag for geographic scope
PolicyNotes	Character	Notes on the policy record
PolicySource	Character	Data source: OxCGRT^45^

**Table 8:** Policy data types.

Type	Description	Type	Description
***CX***	***Containment and closure policies***	***VX***	***Vaccine policies***
C1	School closing	V1	Vaccine prioritization
C2	Workplace closing	V2	Vaccine availability
C3	Cancel public events	V2A	Vaccine availability (summary)
C4	Restrictions on gatherings	V2B	Vaccine availability (age, general)
C5	Close public transport	V2C	Vaccine availability (age, at risk)
C6	Stay at home requirements	V2D	Vaccine availability (medically)
C7	Restrictions on internal movement	V2E	Vaccine availability (education)
C8	International travel controls	V2F	Vaccine availability (frontline)
***EX***	***Economic policies***	V2G	Vaccine availability (healthcare)
E1	Income support	V3	Vaccina financial support
E2	Debt/contract relief	***IX***	***Policy indices***
E3	Fiscal measures	I1	Containment health index
E4	International support	I2	Economic support index
***HX***	***Health system policies***	I3	Government response index
H1	Public information campaigns	I4	Stringency index
H2	Testing policy	***IC***	***Confirmed cases***
H3	Contact tracing	***ID***	***Confirmed deaths***
H4	Emergency investment in healthcare	IXD	*Policy indices (Display)*
H5	Investment in vaccines	IXL	*Policy indices (Legacy)*
H6	Investment in vaccines	IXLD	*Policy indices (Legacy, Display)*
H7	Vaccination policy	IXS	*Policy indices (Simple Average)*
H8	Protection of elderly people	IXW	*Policy indices (Weighted Average)*
***MX***	***Miscellaneous policies***		
M1	Wildcard		

### Other data

#### Prevalence of comorbid conditions

National-level data and United States administrative level 1 data on the prevalence of underlying health conditions associated with increased risk of COVID-19 morbidity and mortality as defined by the Centers for Disease Control and Prevention (CDC) described in Table 9 were compiled from multiple sources. These comorbid conditions included prevalence of human immunodeficiency virus (HIV) infection, obesity, hypertension, smoking, chronic obstructive pulmonary disease (COPD), and cardiovascular disease (CVD)^47^. In addition, national-level indicators of the proportion of the population at increased risk for COVID-19 due to comorbid conditions were compiled from the estimates of Clark and colleagues and included in the unified database^48^. Data was collected from sources online associated with reputable health organizations, health research centers, international and national organizations, research journals, and academic institutions^48–58^. Once compiled, the final data structure was created in Microsoft Excel with all corresponding and available data.

**Table 9:** Static health data structure.

Column	Description
Diabetes^49^,^50^	Age-adjusted percent prevalence of adults with diabetes
Obesity^51^–^53^	Percent of obese adults (body mass index of 30+)
Smoking^54^,^55^	Age-adjusted percent prevalence of adults who are smokers
COPD^56^	Age-standardized percent prevalence of chronic obstructive pulmonary disease by sex
COPD_F^56^	Age-standardized percent prevalence of chronic obstructive pulmonary disease (Female)
COPD_M^56^	Age-standardized percent prevalence of chronic obstructive pulmonary disease (Male)
CVD^56^	Age-standardized percent prevalence of CVD by sex
CVD_F^56^	Age-standardized percent prevalence of CVD (Female)
CVD_M^56^	Age-standardized percent prevalence of CVD (Male)
HIV^56^	Age-standardized percent prevalence of HIV/AIDS by sex
HIV_F^56^	Age-standardized percent prevalence of HIV/AIDS (Female)
HIV_M^56^	Age-standardized percent prevalence of HIV/AIDS (Male)
Hypertension^57^,^58^	Percent of adults with hypertension by sex (Total)
Hypertension_F^56^	Percent of adults with hypertension by sex (Female)
Hypertension_M^56^	Percent of adults with hypertension by sex (Male)
Risk_Tot^47^	Proportion of individuals in the population that have at least 1 of the 11 identified risk conditions for COVID-19.
Risk_Age^47^	Age-standardized proportion of the population that are at increased risk for COVID-19.
Risk_High^47^	Proportion of individuals at high risk, defined as those that would require hospital admission if infected.
Cases_MERS^59^	Total MERS cases by country (October 2012 - Feb2018)
Cases_SARS^60^	Total SARS cases by country (1 Nov 2002 - 7 Aug 2003)

#### Pandemic preparedness

National numbers of cases from the SARS-CoV-1 and MERS outbreaks, as described in Table 9, were included in the unified database as proxy indicators of pandemic experience, which may be relevant for preparedness^59,60^.

#### Accessibility to cities and healthcare facilities

Population-level access to healthcare and other infrastructure may affect the trajectory of pandemics at a local scale by influencing contact rates and the introduction of new infected and susceptible individuals, as well as the speed and likelihood with which new cases are confirmed, treated, and registered in health information systems. Table 10 lists three indicators of accessibility that are included in the unified dataset. Accessibility to nearest cities through surface transport (**Access_City**), quantified as minutes required for traveling one meter, was obtained by extracting zonal statistics from the “Accessibility to Cities 2015” raster file provided by the Malaria Atlas Project (MAP)^61^. The raster file represents the fastest traveling speed from any given point to its nearest city. It was calculated by mapping the travel time at different spatial locations and topographical conditions into grids where the fastest mode of transport took precedence^62^. Using a similar methodology, Weiss and colleagues utilized data from OpenStreetMap, Google Maps, and academic researchers to produce maps of travel time to health care facilities with and without access to motorized transport, from which we obtained the two variables characterizing travel time (minutes) to the nearest healthcare facility by two modes of transport (**Access_Motor**: motorized transport available; **Access_Walk**: no access to motorized transport) as indicators of healthcare access^63^. While country-specific estimates of comparable accessibility metrics exist^64,65^ and may in some cases offer advantages over the global MAP products, we prioritized the latter for its completeness of coverage and standardized methodology, which offers greater comparability across regions and countries.

**Table 10:** Static air quality, accessibility, and population data structure.

Column	Description
ID	Geospatial ID, unique identifier
PM2.5^42^	Fine particulate matter (PM2.5; µg/m^3^) concentration (2014–2018 mean)
PM2.5_PopWtd^42^,^66^	Fine particulate matter (PM2.5; µg/m^3^) concentration (2014–2018 mean, population weighted)
NO2^44^,^45^	Nitrogen dioxide (NO2; ppbv) concentration (2014–2018 mean)
NO2_PopWtd^44^,^45^,^66^	Nitrogen dioxide (NO2; ppbv) concentration (2014–2018 mean, population weighted)
Access_City^61^,^62^	Travel time to nearest cities (minutes)
Access_Motor^63^	Travel time to health care facilities, with motorized transport (minutes)
Access_Walk^63^	Travel time to health care facilities, without motorized transport (minutes)
WorldPop^66^	Total population from WorldPop
WorldPop_Density^66^	Population density from WorldPop (1/km^2^)
WorldPop_65^66^	Population over 65 years old from WorldPop
WorldPop_F^66^	Population by sex (Female) from WorldPop
WorldPop_M^66^	Population by sex (Male) from WorldPop
Sex_Ratio^66^	Sex ratio (Male/Female) from WorldPop

#### Population density and age structure

Table 10 describes population density and age structure from WorldPop^66^.

Total population (**WorldPop**), population density (**WorldPop_Density**), the total population over 65 years old (**WorldPop_65**), and total population by both male (**WorldPop_M**) and female (**WorldPop_F**) were obtained by extracting zonal statistics with the 2020 unconstrained global mosaics raster files at 1 km resolution from the WorldPop spatial datasets, an open access harmonized set of gridded geospatial layers with global coverage produced by drawing on census, survey, satellite and cell phone data. The ratio of male-to-female population (**Sex_Ratio**) was calculated by dividing the female population by male population.

### Data sources

The data sources are listed in Table 11.

**Table 11:** Data sources of the unified COVID-19 dataset.

Source	Description	Level
JHU^1^	Johns Hopkins University Center for Systems Science and Engineering (CSSE)	Global & County/State, United States (US)
CTP^2^	The COVID Tracking Project	State, US
NYC^3^	New York City Department of Health and Mental Hygiene	ZCTA/Borough, New York City
NYT^4^	The New York Times	County/State, US
SES^5^	Monitoring COVID-19 Cases and Deaths in Brazil	Municipality/State/Country, Brazil
DPC^6^	Italian Civil Protection Department	NUTS 0-3, Italy
RKI^7^	Robert Koch-Institut, Germany	NUTS 0-3, Germany
JRC^8^	Joint Research Centre	Global & NUTS 0-3, Europe
IHME^13^	Institute for Health Metrics and Evaluation	National (global) & subnational (US)
CRC^28^	Johns Hopkins Centers for Civic Impact	National (global) & subnational (US)
CIESIN^35^	Center for International Earth Science Information Network	All levels
NLDAS^36^	North American Land Data Assimilation System	County/State, US
ERA5^37^	The fifth generation of ECMWF reanalysis	All levels
Hammer^42^	Fine Particulate Matter Concentrations	All levels
Anenberg^44^,^45^	Global surface NO2 concentrations 1990-2020	All levels
OxCGRT^46^	Oxford COVID-19 Government Response Tracker	Global & subnational (US, UK)
Clark^48^	Lancet estimates of population at increased risk of severe COVID-19 in 2020	Global, regional, national
MAP^61^–^63^	Accessibility to Cities, Accessibility to Healthcare	All levels
WorldPop^66^	Open-Source Demographic Data and Research	All levels

## Technical Validation

The unified data are regularly validated before and after processing by checking and comparing all fields with the available authoritative data sources, such as the World Health Organization (WHO), the US and European Centers for Disease Control and Prevention (CDC), and between the different sources^9–11^. Any significant discrepancy or unrealistic data (e.g., bad data fields or types, negative counts, and implausible values) are automatically detected by checking the type of the data fields (e.g. integer, double, character, or date) and rate of daily changes to investigate and correct the unified data, besides the JHU CSSE’s automatic anomaly detection system, which is designed to detect abrupt spikes or negative increases of daily cases counts. The anomaly detection and data corrections are grouped by geospatial ID, considering recent trends and total population, and data source. Moreover, the geospatial IDs are verified with the corresponding ISO codes and shapefiles for all geographic units. All components of the dataset are updated daily to sync all retrospective changes from the original sources, including any corrections or re-assignments of the case counts. The updated dataset offers more accurate and up-to-date information for researchers to model and analyze COVID-19 transmission dynamics and associations with environmental conditions.

Hydrometeorology and air quality data are all drawn from data sources that perform their own extensive evaluation routines. We did not apply additional independent evaluation of these products. Processed variables were checked for consistency with the source data to ensure that no artifacts were introduced during data transfer or spatial averaging. We perform regular checks of time-series hydrometeorological data from select administrative units in order to scan for inconsistencies or discontinuities in the ERA5 or NLDAS data records, as such errors can sometimes appear in operational Earth data products. To date we have not identified any problematic issues, but should they arise, those data will be flagged as preliminary until corrected versions of the hydrometeorological data files are posted by the operational data center.

The accessibility to cities, validated by comparing it to the network distance algorithm within Google Maps, was encouraging (R^2^ = 0.66; mean absolute difference 20.7 min). The prevalence of comorbid conditions as outlined in Table 9 were taken from online sources directly or associated with reputable health organizations, health research centers, international and national organizations, research journals, and academic institutions. Multiple validation checks were conducted to ensure that our unified dataset matches these input sources. Pandemic preparedness data were taken from similarly internationally-recognized research institutions and global health organizations. Multiple validation checks were conducted to ensure consistency between the unified datasets and these highly vetted data sources.

## Usage Notes

Some US counties, territories, and islands do not have standard FIPS codes or are combined from standard units such as Bristol Bay plus Lake and Peninsula Borough, Dukes and Nantucket counties, Utah jurisdictions, Federal Correctional Institution (FCI), Veterans’ Affairs, and Michigan Department of Corrections (MDOC). Those units are given a unique ID as listed in the frequently-updated lookup table on GitHub.

The Covid Tracking Project (CTP) data stopped updating on March 7, 2021, after one year of service^2^. All other time-varying sources are currently updated/synced from the original sources on a daily basis.

The daily new cases for some units might be missing or negative when calculated from the total accumulated cases in the raw data. This can be attributed to reporting issues and reassignment of the cases. We correct and validate the data entries only when we have strong evidence to do so. Otherwise, we keep the original data exactly as obtained from the official sources. In the future, we plan to provide an augmented version of the global data at all administrative levels, derived from all data sources. Here, we maintain consistency between both the unified and raw data.

The short lifetime of PM_2.5_ and NO_2_ and the spatial heterogeneities in their emissions sources can result in substantial differences between simple and population weighted averages at times, depending on the spatial distribution of the population and emission sources within administrative units. Due to limited availability of ground monitors in some locations, the NO_2_ concentrations have greater certainty in urban areas compared with rural areas and in North America and Europe compared with other parts of the world^44^.

The population by sex data were entered as missing values for thirty-four subnational areas in Brazil since reported values were incompatible with the total population. The accessibility raster file did not cover Monaco, and the data were manually entered using values in the surrounding area. We exclude small, overseas NUTS administrative divisions (e.g., Guadeloupe, French Guiana, Réunion) from the unified dataset to decrease the computational time needed to update the dataset in near real-time. Of note, the accessibility and population data would be most relevant for analysis at subnational, rather than national level, due to the operational definition of the data.

We claim that the presentation of material therein does not imply the expression of any opinion whatsoever on the part of JHU concerning the legal status of any country, area or territory or of its authorities. The depiction and use of boundaries, geographic names and related data shown on maps and included in lists, tables, documents, and databases on this website are not warranted to be error free nor do they necessarily imply official endorsement or acceptance by JHU.

## Data Format

The data are stored in multiple compressed data formats: RDS and FST binary data files supported by R Statistical Software and CSV data files supported by all other machine learning tools. The R binary data formats efficiently preserve all variable types, attributes and object classes. Moreover, RDS files are highly compressed making it easier for file transfer and storage while the FST format provides lightning-fast multithreaded data serialization and full random access to stored datasets allowing for loading a data subset (selected columns or rows) without reading the complete data file. This offers an advantage over other common data formats, such as comma-separated values (CSV) or its compressed versions, that do not explicitly specify the variable types (e.g., integer vs double). Moreover, the produced files are much smaller in size, facilitating data access and processing.

## Data Availability

The source code used to clean, unify, aggregate, and merge the different data components from all sources will be available on GitHub at https://github.com/CSSEGISandData/COVID-19_Unified-Dataset.
